# ezAlign: A Tool for Converting Coarse-Grained Molecular Dynamics Structures to Atomistic Resolution for Multiscale Modeling

**DOI:** 10.3390/molecules29153557

**Published:** 2024-07-28

**Authors:** W. F. Drew Bennett, Austen Bernardi, Tugba Nur Ozturk, Helgi I. Ingólfsson, Stephen J. Fox, Delin Sun, C. Mark Maupin

**Affiliations:** 1 Lawrence Livermore National Laboratory, Livermore, CA 94550, USA; bernardi1@llnl.gov (A.B.); ozturk1@llnl.gov (T.N.O.); ingolfsson1@llnl.gov (H.I.I.); sun25@llnl.gov (D.S.); 2 Procter and Gamble, Reading RG2 0RX, UK; fox.sf@pg.com; 3 Procter and Gamble, Mason, OH 45040, USA; mark.maupin@pnnl.gov; 4 Pacific Northwest National Laboratory, Richland, WA 99352, USA

**Keywords:** molecular dynamics, multiscale modeling, software

## Abstract

Soft condensed matter is challenging to study due to the vast time and length scales that are necessary to accurately represent complex systems and capture their underlying physics. Multiscale simulations are necessary to study processes that have disparate time and/or length scales, which abound throughout biology and other complex systems. Herein we present ezAlign, an open-source software for converting coarse-grained molecular dynamics structures to atomistic representation, allowing multiscale modeling of biomolecular systems. The ezAlign v1.1 software package is publicly available for download at github.com/LLNL/ezAlign. Its underlying methodology is based on a simple alignment of an atomistic template molecule, followed by position-restraint energy minimization, which forces the atomistic molecule to adopt a conformation consistent with the coarse-grained molecule. The molecules are then combined, solvated, minimized, and equilibrated with position restraints. Validation of the process was conducted on a pure POPC membrane and compared with other popular methods to construct atomistic membranes. Additional examples, including surfactant self-assembly, membrane proteins, and more complex bacterial and human plasma membrane models, are also presented. By providing these examples, parameter files, code, and an easy-to-follow recipe to add new molecules, this work will aid future multiscale modeling efforts.

## 1. Introduction

There is great interest in using multiscale modeling to study molecular self-assembly, such as the interactions between membranes and amphiphiles. This interest is particularly motivated by the large range of time and length scales that are important for characterizing these systems. For example, individual hydrogen bonds and other fluctuations that are crucial for some bilayer properties are on the sub-nanometer and sub-nanosecond scales [1], while at the other extreme, bilayer properties such as bending and lipid flip-flop can span hundreds of nanometers and the time scale of hours to days, respectively [2]. To this end, lipid systems are studied with a variety of computational methods, from continuum methods for macroscopic simulations to quantum calculations on extremely small scales. In between these extremes are atomist (AA) and coarse-grained (CG) molecular dynamics (MD) simulations. CG simulations lack atomistic information but retain sub-molecular resolution and can reach orders of magnitude larger and longer simulations than AA simulations.

**Figure 1:**
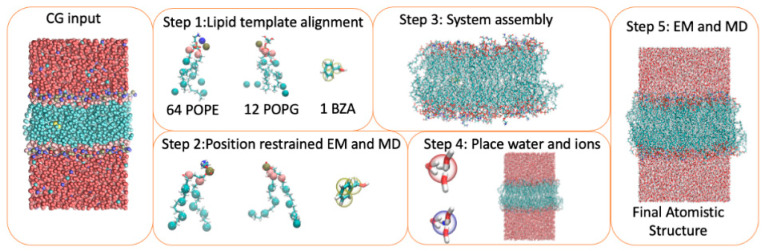
Schematic showing the ezAlign protocol. Starting on the left panel with a CG system for back mapping, each AA molecule is independently aligned to its CG counterpart (Step 1). The CG beads are used for the reference positions for position restraints of the mapped atoms, and a series of energy minimization and stochastic dynamics are run to allow each AA molecule to adopt a conformation consistent with the CG molecule (Step 2). Lipids and small molecules are then combined and relaxed through interactions (Step 3). Finally, water and ions are placed according to the 4–1 mapping of AA-CG waters and four waters in each ion’s solvation shell (Step 4). Short minimization and equilibration steps are used to relax the system and release the position restraints (Step 5).

Converting from a CG structure to an AA representation is a non-trivial task, as one CG particle represents multiple atoms. Therefore, it is not straightforward to place all the atoms inside the CG bead. Figure 1 illustrates the two resolutions, showing a single CG lipid, its corresponding AA lipid, as well as a complex membrane system for each resolution. Multiscale modeling, where both AA and CG models are used in conjunction with one another, offers a great opportunity for obtaining the best of both methods, bridging the micro- and macro-scales. To aid these studies, a number of tools have been built that convert CG to AA models for MD simulations, both from decades ago [3,4] and a number of recent methods [5,6,7]. Backward is one of the most widely used CG-to-AA tools [5]. This method uses pre-defined geometrical relationships for all the atoms in a molecule to place them relative to the CG beads. This is followed by a series of position-restrained energy minimizations and a short MD simulation to relax the system. With this method and any other fragment-based/geometrical method, care must be taken, particularly with regards to unphysically stretched bonds and the chirality of molecules. After back mapping, some bonds within a lipid molecule may be stretched out significantly, which can result in AA simulation systems that are difficult to equilibrate. In such cases, a lipid tail can pierce through an aromatic ring belonging to a membrane protein (called ring penetration in the CHARMM GUI). Our method includes a post-conversion protocol consisting of position-restrained energy minimization and molecular dynamics steps, so that the resulting AA models can be easily equilibrated and simulated. Another important problem is that some molecules can have incorrect stereochemistry after back mapping. Other fragment-based tools include CG2AT [6] and CG2AT2 [7]. Additionally, for fragment-based/geometrical methods, adding new molecules can require careful testing, additional parameterization, and specific chemical knowledge.

Aiming for simplicity and automatability, we implemented ezAlign, a simple, template-based back-mapping tool that requires minimal human time and intervention. This method uses GROMACS [8] and MDAnalysis [9], and similarly to CGTools, developed by Schulten and coworkers in NAMD [4], the initial back mapping of a CG molecule is performed based on an alignment between a subset of individual atoms and their corresponding CG beads. In Figure 1, we summarize the method using a membrane system, where first a template AA molecule is fitted onto each CG lipid using the RMSD of the CG beads to the mapped atoms, utilizing the MDAnalysis Python package [9]. A position restraint is then placed on each mapped atom, with the reference position set by the corresponding CG bead’s position. Each lipid is then energy-minimized without intermolecular interactions, followed by a short stochastic MD simulation. Figure 1 shows a single POPC molecule after using ezAlign, where the AA lipid is found to closely match that of the CG lipid. Four AA waters and ions with four additional waters are then mapped onto each CG water bead, which is similar to the Backward approach for water [5]. The entire system is then assembled, energy is minimized, and a short, restrained MD simulation is conducted, resulting in a full AA system that is ready for subsequent MD simulations. We assess ezAlign’s performance on a simple POPC bilayer compared with Backward and CHARMM-GUI’s Martini to All Atom Converter, followed by presenting several example applications of more complex systems.

## 2. Results

### 2.1. POPC Lipid Bilayer

**Figure 2:**
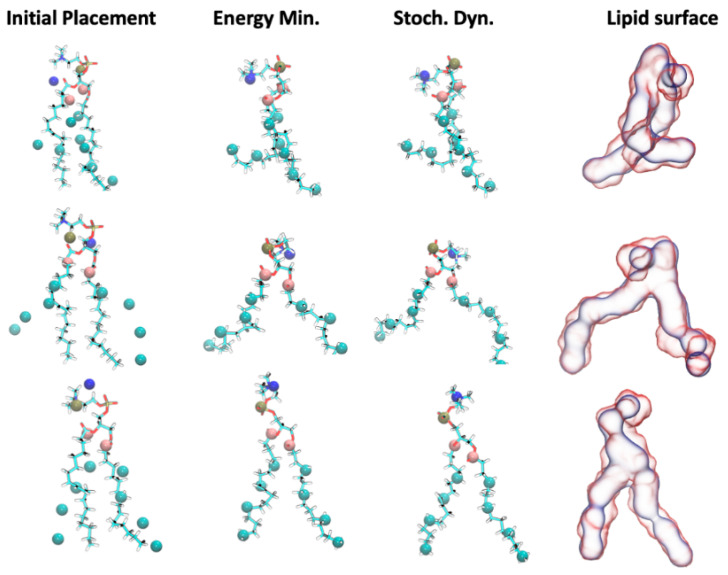
Single POPC lipid conformations during the ezAlign procedure. Starting on the left, a single atomistic lipid conformation (i.e., same for each row) is fit to the respective CG lipid. After energy minimization, the AA atoms overlap with the mapped CG bead, and a short-position restrained stochastic dynamics simulation improves the lipid conformations. The final column on the right overlaps the CG lipid surface (blue) with the AA lipid surface (red) after ezAlign.

Initial testing was performed with a pure POPC lipid bilayer system, which was built with an *insane* bilayer builder [10], as explained in Section 4.4. A 200 ns CG MD simulation with the Martini 2.0 force field was then conducted, and ezAlign was used to convert the final simulation frame to an AA representation using the CHARMM36 force field [11]. Figure 2 shows examples of three lipids during the ezAlign procedure. After the initial placement, each atomistic POPC lipid has the same conformation. Energy minimization with position restraints results in the lipids adopting conformations with the mapped atoms closely overlapping the CG model. An additional step of stochastic dynamics with position restraints results in more relaxed conformations.

### 2.2. Comparison to Backward and CHARMM-GUI

**Figure 3:**
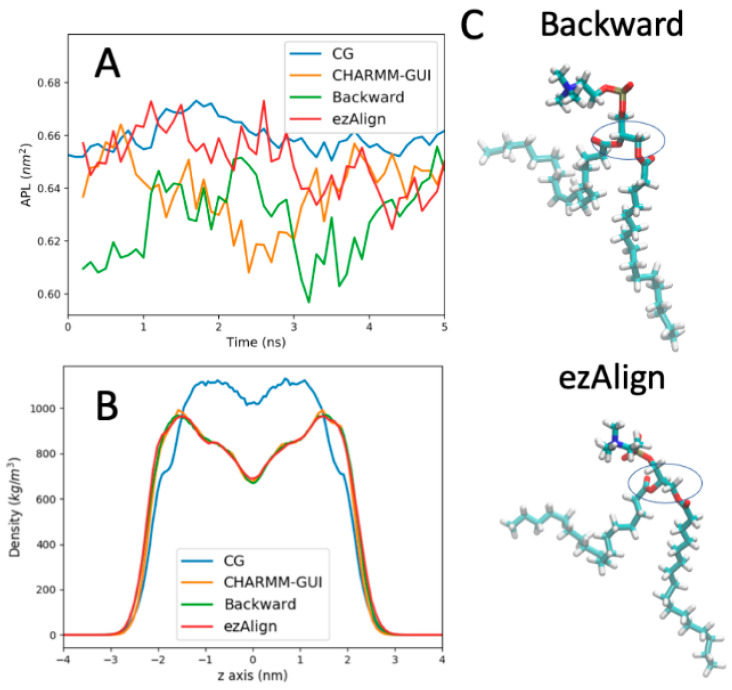
Comparing methods to set up AA MD simulations of a POPC lipid bilayer. (**A**) Area per lipid (nm^2^) following the conversion from CG to AA for Backward and ezAlign and following equilibration for the CHARMM-GUI setup and CG system. (**B**) Density profiles for POPC lipids in the bilayer averaged over 5 ns of simulation time. (**C**) A single POPC lipid that was back mapped with Backward and ezAlign. For this single lipid, the Backward method flipped the chirality of the glycerol backbone (encircled above), while ezAlign maintains the correct chirality for POPC.

To validate ezAlign, we compared both AA simulation setups with CHARMM-GUI’s membrane builder [12] and CG to AA mapped simulations using Backward [5]. For both ezAlign and Backward, the system was initiated from a CG POPC lipid bilayer built with *insane* [10]. Figure 3A plots the area per lipid (APL) for the system at the start of the simulation. All three methods start within the expected range of APL fluctuations, which are close to the CG APL. In Figure 3B, we also compare partial density curves for POPC after conversion to atomistic detail and 5 ns of simulation. These data show that all three methods can produce equilibrated AA starting structures for simple membrane systems.

It has been noted previously that Backward occasionally produce molecules that have a different chirality than expected. We found one such example for POPC in Figure 3C, where the glycerol backbone for the POPC lipid is opposite to the expected state shown for the same lipid converted with ezAlign. We note that this behavior in Backward is rare, and additional restraints or other geometric rules could be added to ensure chirality.

### 2.3. Self-Assembly 

**Figure 4:**
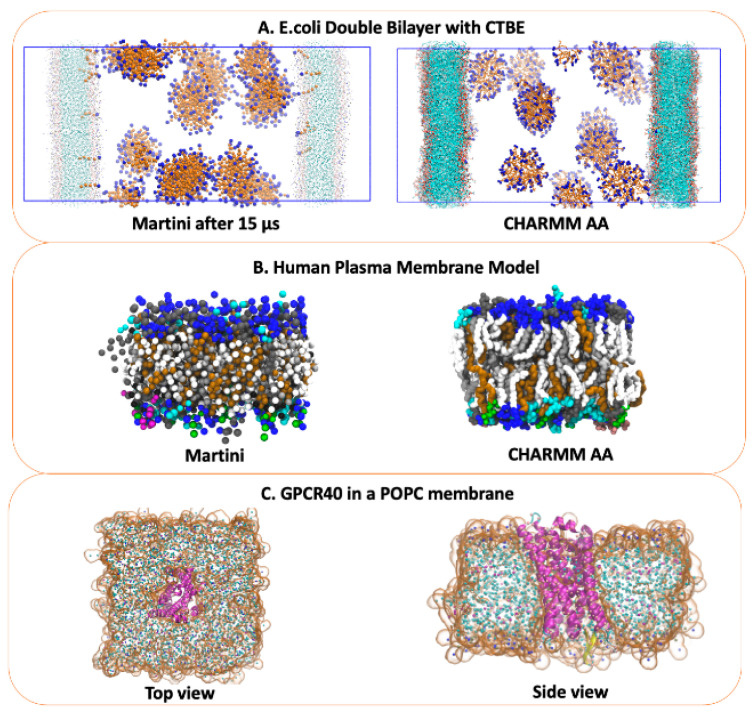
(**A**) CG (**left**) and ezAlign AA (**right**) amphiphilic molecular self-assembly and interaction with an *E. coli* inner membrane model. The CTBE tails are orange, and head groups are blue. The POPE and POPG lipids are colored by atom type. The atomistic system contains over 600,000 atoms. (**B**) Complex model of a human plasma membrane run first with CG (**left panel**) and converted to AA with ezAlign. Water and ions are not shown for clarity. (**C**) GPR40 protein in a POPC lipid bilayer converted from CG to AA using ezAlign, showing a top-view (**left**) and side-view (**right**). The AA protein is colored magenta, with the CG backbone beads as pink spheres. The CG POPC lipid bilayer is also represented with spheres, colored by bead type. The AA lipids are shown as an orange, semi-transparent surface. Water is not shown for clarity.

The self-assembly of amphiphilic molecules is important for many diverse applications, from biotechnology (drug delivery) to chemical engineering (soap formulations) [13]. Due to the necessarily large length and time scales, simulating self-assembly with AA models is challenging. ezAlign is not restricted to membrane systems and is applicable to self-assembly systems. Figure 4A shows an example system of a cetyl-betaine (CTBE) self-assembled into small spherical micelles with long-time scale CG simulations and back mapped with ezAlign. The micelles interact with a model for an *E. coli* inner membrane model on a long timescale, with contacts lasting microseconds of simulation time. Over the long-timescale CG simulations, molecular rearrangements are possible, such as monomer CTBE molecules moving from micelles to the *E. coli* membrane. Figure 4B illustrates a large system that was first run with CG and then back mapped to AA with ezAlign. Large molecular rearrangements are observed, and the collective behavior can then be assessed at both AA and CG levels of detail. In the case of large systems, ezAlign back mapping can take a significant amount of time, but it is still orders of magnitude shorter than standard atomistic production simulations.

### 2.4. Heterogeneous Membranes

We provide several systems for future use that we have mapped, including AA and CG parameters and starting configurations. Cholesterol is an important eukaryotic lipid that has diverse roles in biology and has been studied extensively in membrane simulations [14,15]. Recently, complex models for a human plasma membrane were compared with models with a smaller number of lipid types, resulting in a simplified plasma membrane model consisting of eight lipid types asymmetrically distributed across the leaflets (Mix8) [16]. Figure 4B presents Mix8, showing the complex mixture of lipids that are present in this system, including bilayer asymmetry. Bacterial membranes may also be modeled, such as the *E. coli* cytoplasmic membranes shown in Figure 4A.

### 2.5. Transmembrane Proteins

Transmembrane proteins are another important and well-studied biological system [17]. Simulation systems composed of transmembrane proteins can be quite large and require significant sampling times to adequately establish the lipid/protein interaction ensemble, which are currently inaccessible to AA simulation strategies. With ezAlign, ensemble equilibration can first be performed in CG and then back mapped to AA with ezAlign, yielding significantly faster lipid/protein interaction equilibration while recovering AA resolution. Figure 4C shows an example where GPR40 is first run with the CG model position restraints to allow the lipids to equilibrate. The 200 ns CG frame was then converted to AA using ezAlign. We highlight that with ezAlign, the lipids are packed around the protein, as expected from the CG model.

**Figure 5:**
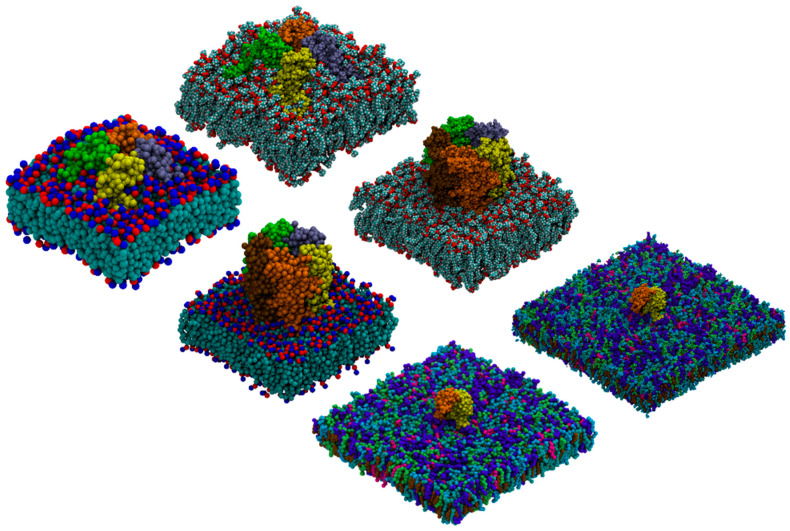
CG (**left**) and AA (**right**) representations of a hERG ion channel in POPC (**top**), a GABA_A_ receptor in POPC (**middle**), and a RAS/RAF complex in a human plasma membrane model (**bottom**). AA systems are back mapped from CG with ezAlign. Solvating water and ions omitted for visual clarity.

In addition to GPCR40, ezAlign has been successfully tested on three other transmembrane protein systems. Figure 5 shows the CG and back-mapped AA representations of hERG [18], GABA_A_ [19], and a RAS/RAF complex [20]. All systems were simulated for 200 ns in CG and then converted to AA resolution with ezAlign. All systems exhibited proper lipid packing around the back-mapped proteins. The RAS/RAF system features a large, complex, heterogeneous human plasma membrane model. This system contains approximately one million atoms, which were readily back mapped with ezAlign in a little less than an hour using 36 CPUs with MPI parallelization.

## 3. Discussion

CG structures are readily converted to AA resolution for multiscale simulations with the new tool ezAlign, publicly available at github.com/LLNL/ezAlign. The ezAlign program is easy to use and allows for the accurate transformation of CG systems to AA detail, where each AA molecule matches its corresponding CG molecule’s conformation. Currently, ezAlign is readily capable of converting several standard molecules with no user modifications, including biologically relevant systems such as lipid membranes, proteins, and small molecules. Adding new molecules is a trivial task due to the use of an initial template molecule. A similar method was previously implemented in NAMD and shown to effectively convert the CG structure to AA representation [4] and applied to a number of interesting problems [21,22] and extensions to polymers [23]. Our tool is implemented for use with GROMACS [8], has a wealth of pre-built systems, and is easily extendable to other systems. Our code uses MDAnalysis [9] for the molecule transformations, so adapting to another MD code or other force fields is, in principle, straightforward. 

The ezAlign program reproduces the structure of a lipid bilayer with similar accuracy and efficiency as the popular Backward tool [5]. One advantage of ezAlign is that it is very straightforward to add new molecules. Additionally, Backward and other geometry-based tools can result in molecules with the wrong chirality. Due to the form of the AA MD potential energy function, these states are permissible but change the molecules chemistry, possibly in important ways. Backward has additional parameters that can be added to prevent improper placement or dihedral potentials enforcing a specific tautomer, but these methods require prior chemical knowledge. Other fragment- or geometric rule-based methods, such as CG2AA [7], will also likely suffer from this deficiency. Recently, machine learning tools for CG-to-AA transformations have been developed [24]. These tools require extensive training data, which requires significant work to produce for each new molecule and system. The transferability of the ML model to other situations is also a potential problem. For example, training how to back map a molecule in water is likely not suitable if the molecule is in a lipid membrane.

Systems including a protein complex may be modeled with ezAlign, including transmembrane protein systems. Particularly for membrane proteins that are either known or expected to deform the bilayer morphology, an MD simulation system at CG resolution is often easier to build and equilibrate, even if the simulations themselves are to be carried out at AA resolution. Once the membrane solvation around the protein is equilibrated well in a CG MD, the system of interest can then be converted to AA representation using ezAlign with ease and efficiency. Apart from alleviating issues regarding the building and equilibrating of complex membrane protein simulation systems, CG simulations of membrane proteins can efficiently achieve an equilibrated distribution of different lipid species within a complex membrane. After a complex protein-lipid system equilibrates the long-timescale protein/lipid interactions with CG resolution, ezAlign can be used to recover AA resolution through back mapping. In addition to its ease of use, ezAlign is easy to modify for specific purposes. For example, the incorporation of nonstandard amino acids is straightforward, requiring no direct modification of the core ezAlign code (see Section 4.2).

There are many avenues for future improvements to ezAlign. The ezAlign program is designed so it can readily be adapted to take advantage of improvements to hardware and software for MD simulation speeds in the future. There is considerable room for optimizing the speed and computational cost of the program. Accommodating more sophisticated mapping strategies could prove useful; as it stands, ezAlign must map CG beads to AA atoms. Finally, expanding to other types of molecules, such as DNA and RNA, can be achieved in future versions. We also plan to expand our list of molecules and pre-equilibrated membranes. 

As simulation capabilities expand, the need for easy access to model systems and multiscale software will expand as well. We provide several diverse applications and parameters for community use with ezAlign. Systems include basic bilayer systems, complex mixtures, bacterial inner membranes, human plasma membranes, and surfactant self-assembly. Multiscale workflows allow for complex mixtures and molecular rearrangements that are difficult to produce with tools such as CHARMM-GUI alone [25]. 

## 4. Methods

### 4.1. ezAlign Protocol

Figure 1 illustrates the steps to convert a pure POPC lipid bilayer system from the CG structure to the AA model. In Step 1, each CG lipid is aligned to a single AA lipid of the same type. A predefined mapping of each bead to a single AA atom is used to define the position restraints for the AA lipid. In Step 2, each lipid is then subjected to a short energy minimization with the AA-mapped atoms restrained to the position of the respective CG bead. After minimization, a short stochastic dynamics simulation is carried out for each lipid in vacuum. If a protein is included, additional protein minimization and relaxation simulations are performed in vacuum (see Section 4.2). In Step 3, the system is constructed by merging all the lipids, proteins, and other molecules. In Step 4, water (four waters per bead) and ions (one ion and four waters to mimic the ‘solvated’ ion paradigm in Martini) are added to the system. In Step 5, the final system is then energy-minimized and equilibrated with position restraints to generate an output AA structure ready for simulation.

### 4.2. Protein Minimization and Relaxation

Most Martini CG protein simulations involve a tight elastic network to maintain secondary and tertiary structure. However, there will be some conformational flexibility as well as sidechain motion. Additionally, future Martini CG protein simulations look to do away with the elastic networks, increasing flexibility and the ability to model conformational dynamics with Go-like models [26]. With ezAlign, each amino acid is mapped according to “amino_map.py” in the “files” directory. This file can be easily modified to permit the incorporation of nonstandard amino acids without any modification of the core ezAlign.py code. Using this by-residue mapping strategy, the protein is minimized and relaxed in vacuum, in an analogous manner to the lipid protocol, such that the relaxed AA protein adopts the same conformation as the CG input system (see “em1_prot.mdp” and “md1_prot.mdp” in the “files” directory for specific parameters). Multiple independent protein complexes can be provided for simultaneous mapping using this protocol.

### 4.3. File Structures

**Figure 6:**
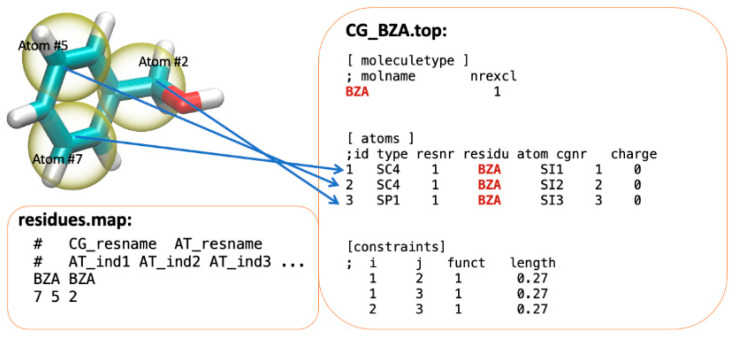
An example CG-to-AA mapping of a benzyl-alcohol molecule. The “residues.map” file contains the mapping of CG beads to each atom. In this example, CG beads 1, 2, and 3 map to AA atoms 7, 5, and 2, respectively. The “CG_BZA.top” file is the CG GROMACS molecular topology.

There are two files that currently must be supplied by the user for each new molecule type that is not already included in the ezAlign “files” subdirectory. The atomistic force-field files (in the itp format) must be present, as must the energy-minimized AA PDB files for each molecule type in the simulated system. The file names should match the name of the molecule, with Figure 6 illustrating the file structures, names, and an example mapping for a small three-bead benzyl alcohol molecule.

When running ezAlign, an initial CG structure for a large system with many molecules must be provided as a PDB file with a corresponding CG topology file, which maps the number of molecules of each type. The “residues.map” file in the “files” subdirectory must be modified for the inclusion of new molecules, using the format illustrated in Figure 6. 

### 4.4. Coarse-Grained MD Simulations

A pure POPC lipid membrane was built with the insane bilayer builder [10] and solvated with 0.15 M salt solution. Martini 2.0 parameters were used to calculate the bonded and non-bonded interactions. Ten percent of water beads were modeled as anti-freeze water beads (WF), whereas the rest were modeled as regular water beads (W). For the initial CG setup and runs, we used the Martini v2.0 force field [27] and the *insane* bilayer builder [10]. MD simulations were run with a 20 fs time step in GROMACS 2018.3 and 5.1.4 [8]. 

Temperature was maintained at 313 K using the V-rescale method [28], and semi-isotropic pressure coupling was used with the Parrinello-Rahman method [29] and 1 bar pressure. Non-bonded interactions were cut off after 1.2 nm. For electrostatic interactions, a dielectric of 15 is used for implicit charge screening and is shifted from 0 nm to 1.2 nm. Lennard-Jones interactions were shifted from 0.9 nm to 1.2 nm.

### 4.5. Atomistic MD Simulations

Atomistic simulations were run with GROMACS 2018.3 and GROMACS 2023.2 [8]. Note the current version of ezAlign requires a GROMACS version later than 2022, due to the utilization of the Gapsys et al. soft-core potential [30]. A time step of 2 fs was used with LINCS constraints on the hydrogen bonds and angles [31,32]. Lennard-Jones interactions were cut off at 1.0 nm, and long-range electrostatic interactions were computed using the particle mesh Ewald method [33,34]. Semi-isotropic pressure coupling was used with the Parrinello-Rahman [29] barostat with a reference pressure of 1 bar. Temperature was maintained at 313 K using the Nose-Hoover method [35].

### 4.6. Transmembrane Protein Simulations

We also simulated the transmembrane protein GPR40 (PDB: 4EJ4), starting from the x-ray structure [36]. For Martini, the v2.2 model was used for the protein [37] and v2.0 for the lipids [27], with simulation parameters the same as for the bilayer-only models. The system was then converted into AA with ezAlign, using the AMBER99 force field [38] for the protein and the AMBER21 lipids [39]. This system was then run for 50 ns to monitor stability. The lipid surface density was calculated and plotted using VMD [40].

Transmembrane proteins hERG (PDB: 7CN1) [18], GABA_A_ (PDB: 8SI9) [19], and a RAS/RAF complex (PDB: 6XI7) [20] were also simulated. The same protocol was used as GPR40, except the CHARMM36 [11] force field was used instead of AMBER.

## 5. Conclusions

The program ezAlign, a new tool for CG-to-AA resolution transformations, is presented for future use by the scientific community. We validated ezAlign against other methods for converting lipid membrane systems from CG to AA resolution. One significant advantage of ezAlign is its ease of use, where adding new molecules is a trivial and automatable task. Additionally, ezAlign does not require training data or human knowledge of chemistry and can back map complex membrane-protein systems. The ezAlign program is provided with files for several important biological systems, including proteins, lipids, and surfactants, for easy adoption by new users.

## Data Availability

The ezAlign source code, examples, and documentation may be found at https://github.com/LLNL/ezAlign (accessed on 27 July 2024).
